# Meropenem versus piperacillin-tazobactam for definitive treatment of bloodstream infections due to ceftriaxone non-susceptible *Escherichia coli* and *Klebsiella* spp (the MERINO trial): study protocol for a randomised controlled trial

**DOI:** 10.1186/s13063-014-0541-9

**Published:** 2015-01-27

**Authors:** Patrick NA Harris, Anton Y Peleg, Jon Iredell, Paul R Ingram, Spiros Miyakis, Andrew J Stewardson, Benjamin A Rogers, Emma S McBryde, Jason A Roberts, Jeff Lipman, Eugene Athan, Sanjoy K Paul, Peter Baker, Tiffany Harris-Brown, David L Paterson

**Affiliations:** University of Queensland Centre for Clinical Research, Building 71/918 Royal Brisbane & Women’s Hospital Campus, Herston, 4029 Brisbane, QLD Australia; Department of Infectious Diseases, The Alfred Hospital, Melbourne, VIC Australia; Westmead Millenium Institute for Medical Research, Westmead Hospital, Sydney, NSW Australia; Department of Microbiology and Infectious Diseases, Royal Perth Hospital, Perth, WA Australia; School of Pathology and Laboratory Medicine, University of Western Australia, Crawley, WA Australia; Department of Infectious Diseases, School of Medicine, University of Wollongong and The Wollongong Hospital, Wollongong, NSW Australia; Department of Infectious Diseases, Austin Health, Melbourne, VIC Australia; Monash Infectious Disease, Monash Health, Clayton, VIC Australia; Victorian Infectious Diseases Unit, Royal Melbourne Hospital, Melbourne, VIC Australia; Burns Trauma and Critical Care Research Centre, The University of Queensland, Brisbane, QLD Australia; Department of Infectious Disease, Barwon Health, Deakin University, Geelong, VIC Australia; Clinical Trials & Biostatistics Unit, QIMR Berghofer Medical Research Institute, Brisbane, QLD Australia; Queensland Clinical Trials and Biostatistics Centre, University of Queensland, Brisbane, QLD Australia

**Keywords:** Extended-spectrum beta-lactamase, ESBL, Plasmid-AmpC, Therapy, Resistance, Beta-lactam/beta-lactamase inhibitor, Carbapenem, Clinical trial

## Abstract

**Background:**

Gram-negative bacteria such as *Escherichia coli* or *Klebsiella* spp. frequently cause bloodstream infections. There has been a worldwide increase in resistance in these species to antibiotics such as third generation cephalosporins, largely driven by the acquisition of extended-spectrum beta-lactamase or plasmid-mediated AmpC enzymes. Carbapenems have been considered the most effective therapy for serious infections caused by such resistant bacteria; however, increased use creates selection pressure for carbapenem resistance, an emerging threat arising predominantly from the dissemination of genes encoding carbapenemases. Recent retrospective data suggest that beta-lactam/beta-lactamase inhibitor combinations, such as piperacillin-tazobactam, may be non-inferior to carbapenems for the treatment of bloodstream infection caused by extended-spectrum beta-lactamase-producers, if susceptible *in vitro*. This study aims to test this hypothesis in an effort to define carbapenem-sparing alternatives for these infections.

**Methods/Design:**

The study will use a multicentre randomised controlled open-label non-inferiority trial design comparing two treatments, meropenem (standard arm) and piperacillin-tazobactam (carbapenem-sparing arm) in adult patients with bacteraemia caused by *E. coli* or *Klebsiella* spp. demonstrating non-susceptibility to third generation cephalosporins. Recruitment is planned to occur in sites across three countries (Australia, New Zealand and Singapore). A total sample size of 454 patients will be required to achieve 80% power to determine non-inferiority with a margin of 5%. Once randomised, definitive treatment will be for a minimum of 4 days, but up to 14 days with total duration determined by treating clinicians. Data describing demographic information, antibiotic use, co-morbid conditions, illness severity, source of infection and other risk factors will be collected. Vital signs, white cell count, use of vasopressors and days to bacteraemia clearance will be recorded up to day 7. The primary outcome measure will be mortality at 30 days, with secondary outcomes including days to clinical and microbiological resolution, microbiological failure or relapse, isolation of a multi-resistant organism or *Clostridium difficile* infection.

**Trial registration:**

The MERINO trial is registered under the Australian New Zealand Clinical Trials Register (ANZCTR), reference number: ACTRN12613000532707 (registered 13 May 2013) and the US National Institute of Health ClinicalTrials.gov register, reference number: NCT02176122 (registered 24 June 2014).

**Electronic supplementary material:**

The online version of this article (doi:10.1186/s13063-014-0541-9) contains supplementary material, which is available to authorized users.

## Background

Bloodstream infections caused by Gram-negative bacteria such as *Escherichia coli* or *Klebsiella* spp. are commonly encountered in clinical practice and may be associated with significant mortality [1]. These bacteria may also acquire genes encoding beta-lactamase enzymes that hydrolyse essential beta-lactam antibiotics, rendering such treatment ineffective. Isolates expressing extended-spectrum beta-lactamase (ESBL) or plasmid-mediated AmpC enzymes are increasingly encountered across the world, in both community- and hospital-onset infections [2]. ESBL- or AmpC-producers are typically resistant to third generation cephalosporins such as ceftriaxone, but susceptible to carbapenems [3]. These bacteria are usually multi-resistant, as beta-lactamase genes are frequently co-located with other resistance mechanisms, leaving few treatment options. Bacteraemia caused by resistant Gram-negative bacteria may carry mortality in excess of those caused by susceptible strains [4]. Thus, defining optimal treatment regimens in these serious infections is of clinical importance. However, when selecting antimicrobial therapy, clinicians must consider both efficacy of the chosen agent and downstream risk such as selective pressure for further antimicrobial resistance.

In observational studies that have been performed to evaluate antibiotic choices for ESBL-producing Enterobacteriaceae, no agent has been shown to significantly surpass carbapenems [5-8]. However, widespread use of carbapenems may cause selection pressure for carbapenem resistance [9,10]. Carbapenem-resistant Gram-negative bacteria present a great therapeutic challenge, with susceptibility often limited to ‘last-line’ antibiotics such as colistin or tigecycline. Some new beta-lactam antibiotics and beta-lactamase inhibitors, which are active against ESBL-, AmpC- and some carbapenemase-producing organisms, are in advanced clinical development [11,12]. However, these antibiotics are likely to be expensive, are not yet on the market and may best be held in reserve for infections without therapeutic alternatives.

The susceptibility of AmpC- and ESBL-producers to piperacillin-tazobactam is less predictable than carbapenems. By definition, ESBLs are inhibited by beta-lactamase inhibitors such as clavulanate or tazobactam [3]. However, *E. coli* or *Klebsiella* spp. may also produce multiple beta-lactamase types, some of which are resistant to inhibition by tazobactam and may not be evident by susceptibility testing alone. There have also been concerns that inoculum effects may overwhelm the activity of beta-lactamase inhibition in infections with a large bacterial burden [13]. Additionally, in some cases outer membrane protein loss may contribute to resistance to tazobactam [14]. By definition, AmpC enzymes are also not well inhibited by tazobactam [15], and plasmid-mediated AmpC expression has been increasingly recognised as a cause of resistance to expanded spectrum cephalosporins in Enterobacteriaceae [15]. However, despite these concerns, approximately 50% or more of ceftriaxone non-susceptible *E. coli* or *Klebsiella* spp. remain susceptible to piperacillin-tazobactam [16]. Yet, until recently, lack of published clinical experience with the use of beta-lactam/beta-lactamase inhibitor (BLBLI) agents for the treatment of ESBL-producers has limited their use. As a result, carbapenems have been perceived as a superior option. In recent years we have witnessed an alarming increase in the prevalence and global dissemination of carbapenem-resistant Gram-negative bacteria, largely driven by the spread of genes encoding carbapenemase enzymes, transmitted on highly mobile plasmids [17]. Thus, defining carbapenem-sparing treatment options for ESBL-producers has become imperative to reduce the selection pressure for carbapenem resistance [10].

No randomised controlled trials (RCTs) have yet been performed comparing different treatment options for third generation cephalosporin-resistant Enterobacteriaceae. The largest observational study with an analysis by treatment outcome was published in 2012 by Rodriguez-Bano *et al*. [18]. They performed a *post hoc* analysis of six prospective cohorts of patients with bacteraemia due to ESBL-producing *E. coli*. Two non-mutually exclusive cohorts (empirical therapy and definitive therapy) were constructed and analysed separately. In both cohorts, carbapenems were not superior to BLBLI combinations, such as piperacillin-tazobactam. Specifically, in the definitive therapy cohort, mortality rates at 30 days were not significantly different - 9.3% for those who received a BLBLI and 16.7% for those who received a carbapenem (*P* > 0.20) [18]. A subsequent meta-analysis of all published studies examining treatment options for bacteraemia caused by ESBL-producers also concluded that BLBLI agents were non-inferior to carbapenems for both definitive (relative risk (RR) 0.52, 95% confidence interval (CI) 0.23 to 1.13) or empirical therapy (RR 0.91, 95% CI 0.66 to 1.25) with regard to all-cause mortality [6]. In contrast, mortality was lower with carbapenems when compared to non-BLBLI antibiotics (for example, quinolones) for both definitive (RR 0.65, 95% CI 0.47 to 0.91) and empirical treatment (RR 0.50, 95% CI 0.33 to 0.77).

Both meropenem and piperacillin-tazobactam are antibiotics that have been widely used in clinical practice for many years. They have proven efficacy in a wide range of infectious syndromes, including severe sepsis, febrile neutropenia, ventilator-associated pneumonia and intra-abdominal sepsis. Both agents are licensed for the treatment of serious infections and are available for routine clinical use in generic formulations. A non-inferiority study design was selected because it is not ethically possible to conduct a placebo-controlled trial and piperacillin-tazobactam is not expected to be superior to meropenem with regard to the primary end-point. Instead, potential benefits relate to minimisation of ‘collateral damage’ in the form of selection for carbapenem-resistant Enterobacteriaceae.

## Methods/Design

The trial protocol was developed by the Gram-negative Working Group of the Australasian Society for Infectious Disease Clinical Research Network (ASID-CRN), in accordance with the CONSORT statement extension for ‘Non-inferiority and Equivalence Trials’ [19].

### Hypothesis

Our hypothesis is that piperacillin-tazobactam is non-inferior to meropenem for the definitive treatment of bloodstream infections due to third generation cephalosporin non-susceptible *E. coli* or *Klebsiella* spp.

### Study design

The study design will be a multicentre randomised controlled non-inferiority open-label phase III trial. Both study drugs (meropenem and piperacillin-tazobactam) will be administered intravenously with standard dosing regimens. The study population will be all adult patients (18 years of age or older, or 21 years or older at Singapore sites) admitted to participating hospitals. Inclusion in the study will be determined by the presence of a bloodstream infection with *E. coli* or *Klebsiella* spp., as defined by at least one positive blood culture from a peripheral blood draw, where the isolate is confirmed to be third generation cephalosporin non-susceptible, but susceptible to piperacillin-tazobactam and meropenem by European Committee for Antimicrobial Susceptibility Testing (EUCAST) standards [20].

### Setting

The trial will be conducted in several centres across Australia, New Zealand and Singapore (see Table 1).

**Table 1:** **Proposed participating sites**

**Country - State**	**Facility**
Australia - Queensland	Royal Brisbane and Women’s Hospital
	St. Andrew’s War Memorial Hospital
	Brisbane Private Hospital
	Princess Alexandra Hospital
	The Mater Hospital
Australia - New South Wales	Westmead Hospital
	Wollongong Hospital
Australia - Victoria	The Alfred Hospital
	Monash Medical Centre
	The Austin Hospital
	Peter MacCallum Cancer Centre
	Dandenong Hospital
	Barwon Health
Australia - Western Australia	Royal Perth Hospital
New Zealand	North Shore Hospital, Auckland
	Middlemore Hospital, Auckland
Singapore	National University Hospital, Singapore
	Tan Tock Seng Hospital, Singapore

### Intervention

Meropenem 1 gram will be administered every 8 hours intravenously or piperacillin-tazobactam 4.5 grams administered every 6 hours intravenously. Each dose will be given over 30 minutes. The study drug is to be administered for a minimum of 4 days and can be given for as long as 14 days. The total duration of therapy will be determined by the treating clinician. Dose adjustment for renal impairment will be made according to the criteria in Table 2. Blinding will not be performed as the two antibiotics have different dosing regimens. Follow-up will be for 30 days post enrolment. Other antimicrobials active against Gram-negative bacilli are excluded in the first 4 days after enrolment, except that trimethoprim/sulfamethoxazole may be continued as *Pneumocystis* prophylaxis.

**Table 2:** **Dose adjustment for study antibiotics**

	**Meropenem**	**Piperacillin-tazobactam**
Creatinine clearance > 50 mL/min	No change	No change
Creatinine clearance 26 to 50 mL/min	1 g every 12 hours	4.5 g every 8 hours
Creatinine clearance 10 to 25 mL/min	500 mg every 12 hours	4.5 g every 12 hours
Creatinine clearance < 10 mL/min	500 mg every 24 hours	4.5 g every 12 hours
Haemodialysis	500 mg every 24 hours and 500 mg after each dialysis	2.25 g every 8 hours and an additional 0.75 g after each dialysis
Peritoneal dialysis	500 mg every 24 hours	2.25 g every 8 hours
Continuous-renal replacement therapy	1 g every 12 hours	4.5 g every 8 hours

### Primary objectives

- To compare the 30-day mortality post bloodstream infection in patients treated with piperacillin-tazobactam or meropenem

### Secondary objectives

- To compare the time to clinical and microbiologic resolution of infection for each regimen, defined as: number of days from randomisation to resolution of fever (temperature > 38.0°C) and leucocytosis (white blood cell count > 12 × 10^9^/L) PLUS sterilisation of blood cultures
- To compare the clinical and microbiologic success of each regimen at day 4 of the intervention, defined as survival PLUS resolution of fever and leucocytosis PLUS sterilisation of blood cultures. All of these criteria will be assessed on day 4, counted from the day of randomisation (day 1) in order to determine a rapid response from the trial drug
- To compare microbiologic resolution of infection, defined as sterility of blood cultures collected on or before day 4
- To compare the risk of relapse with each regimen, defined as growth of the same organism as in the original blood culture after the end of the period of study drug administration but before day 30
- To compare the risk of superinfection with a carbapenem or piperacillin-tazobactam- resistant organism or *Clostridium difficile*, defined as growth of a meropenem or piperacillin-tazobactam-resistant organism from any clinical specimen collected from day 4 of study drug administration to day 30 or a positive *C. difficile* stool test (with the method of testing used according to local laboratory protocol). This endpoint is important since one of the purposes of establishing an alternative to carbapenem therapy is to reduce infections with resistant organisms or *C. difficile*.

### Randomisation

**Figure 1:**
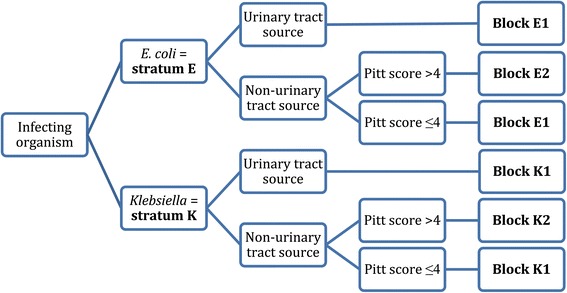
**Patient stratification at enrolment.**

Patients will be randomly assigned to either meropenem or piperacillin-tazobactam in a 1:1 ratio according to a randomisation list prepared in advance for each recruiting site and stratification. Random sequence will be generated using random permuted blocks of unequal length. The randomisation process will be managed by the Queensland Clinical Trials & Biostatistics Centre (QCTBC) of The University of Queensland, and generated using an online data management system (REDCap). Patients will be stratified into 4 groups according to infecting species (*E. coli* or *Klebsiella* spp.) and disease severity (according to a Pitt bacteraemia score [21] ≤ 4 or > 4; and presumed site of infection from the urinary tract or elsewhere) (see Figure 1).

### Safety monitoring plan

A Data Monitoring and Safety Board (DMSB) will be established, comprising two independent infectious disease physicians with statistical support provided to them by the QCTBC. An interim analysis - including both efficacy and safety endpoints - will be performed after the first 50 subjects have completed the 30-day study period. The trial statistician will provide details of safety outcomes and any significant differences in primary outcomes according to treatment arm to the DMSB. The stopping rule would be a statistically significant difference in primary outcomes between the two therapies.

### Data entry and storage

A clinical database using the REDCap trial data management system has been developed with a web hosting facility. Paper case report forms (CRFs) (Additional file 1) will be completed and uploaded to the online system to collect all clinical and additional laboratory-related information (see Table 3). To ensure accurate transcription of the CRF, double-data entry will be performed by a second independent researcher. Any discrepancies, missing data or errors will be clarified by discussion with the site principal investigator. To ensure validity, a proportion of CRFs will be checked by the local site investigators against clinical and laboratory records.

**Table 3:** **Data collection**

	**Variables**
Demographic data	Age, gender, ethnicity, long-term residential care status, ward location
Trial characteristics	Date of screening and enrolment, inclusion criteria and consent details, date and time of randomization
Co-morbidities and risk factors	Charlson score, co-morbid conditions, date and type of any surgery within 14 days, use of cytotoxic chemotherapy, immune suppressive medication, radiotherapy, biological agents (for example, monoclonal antibody therapy), presence of intravascular devices or urinary catheters; use of ‘not for resuscitation’ order
Infection parameters	Bacteraemia acquisition status (community, healthcare-associated or hospital-acquired infection), presumed source of infection, ICU admission, Pitt bacteraemia score, Acute Physiology and Chronic Health Evaluation (APACHE) II score (if in ICU)
Antibiotic data	From 48 hours prior to blood culture collection and up to 30 days; dose/route/frequency recorded
Clinical observations	Daily vital signs, (highest temperature, HR, RR; lowest systolic BP), lowest arterial pCO_2_ (if ventilated), white cell count, use of pressors; recorded days 1 to 7; patient weight
Microbiological data	Date and time of initial blood culture, full susceptibility profile, daily blood culture results days 1 to 3; any further positive blood cultures and species identification/resistance profile; other clinical sites growing *E coli* or *Klebsiella*, any multidrug-resistant organism or *C. difficile* identified up to 30 days
Outcome data	Survival at 7 and 30 days post randomisation
	Date of death or discharge
	Length of hospital stay
	Days to clinical and microbiological resolution
	Clinical and microbiological success at day 4
	Microbiological resolution or relapse
	Protocol violations and adverse events
	Reasons for study withdrawal

*Abbreviations*: *BP* blood pressure, *HR* heart rate, *RR* respiratory rate.

### Determination of sample size

As no randomised clinical trial has yet been conducted in this particular field, the sample size estimation has been derived from the retrospective study of bloodstream infection caused by ESBL-producing *E. coli* performed by Rodriguez-Bano *et al*. [7]. The overall 30-day mortality in their study was 16.7% in those patients who received a carbapenem (our control group). We have conducted a series of simulations with possible variations in the observed rates between the two treatment groups. Considering a mortality rate of 17% in the control group (rounded from the 16.7% actually observed), and a non-inferiority margin of 5% difference in the 2 groups, we would need 280 patients in total to achieve 80% power with a 1-sided alpha level of 0.025. This allows for 10% dropout. It is likely that mortality rates in observational cohorts may be greater than those in a trial with exclusion criteria. Therefore, if the observed mortality rate in the control group was 14% (3% lower than that seen in the observational cohort), then under the same assumptions, we would need 454 patients in total to achieve 80% power [22].

Actual mortality is expected to be similar in our study population. We have performed a retrospective analysis of 92 bacteraemia episodes caused by ceftriaxone-resistant *E. coli* or *Klebsiella* in Singapore (one of the trial sites) over a 12-month period until May 2013, examining patients that would fulfil the MERINO trial inclusion criteria. Average mortality at 30 days was 17.4% in patients given a carbapenem for definitive therapy. However, overall mortality for all treatment groups was lower at 8.7%, including those receiving BLBLIs (8.3%) [23].

### Statistical and analytical plans

The intention-to-treat (ITT) analysis approach, supported by the per-protocol approach, will be adopted to make inference on the possible non-inferiority of the treatment arm, compared to the control arm, in terms of 30-day mortality. The proportions of deaths (with 95% CIs) in the 2 study arms will be calculated. Logistic regression with ‘treatment group’ as the only covariate will be employed to draw inference on the possible non-inferiority of the intervention treatment compared to the control treatment. The odds ratio (with 95% CIs) will be calculated with the meropenem arm as the reference group. Appropriate parametric or non-parametric statistical techniques will be employed to analyse the data for secondary aims of the study. The Mann–Whitney *U*-test will be used to compare median number of days to clinical and microbiological resolution. Logistic regression will be used to compare odds ratios of achieving clinical and microbiological success and microbiological resolution at day 4, as well as the odds ratio for microbiological relapse and superinfection with a resistant organism between each treatment arm. All secondary analyses will be based on an ITT population.

An analysis of primary and secondary endpoints as described above will be undertaken in a subgroup of ‘high risk’ patients defined by a likely source of infection other than the urinary tract or a Pitt bacteraemia score of >4.

Basic statistics in the study report will include information on missing values for all relevant study variables. A summary of baseline patient characteristics with totals and proportions (%) for categorical variables, and minimum, maximum, inter-quartile ranges and standard deviations for continuous variables will be presented. For continuous study variables, box plots and Kernel density plots will also be provided.

### Protocol violations

All protocol violations occurring after randomisation will be listed in the Clinical Study Report, tabulated by subject and recruitment site. The final assignment of participants to the per-protocol analysis population will be made at a blinded protocol violation review meeting prior to database lock.

### Consent

Potential study participants will be identified on the basis of positive blood cultures by liaison between the investigators and the clinical microbiologists. All patients who fulfill the microbiological inclusion criteria will be screened for eligibility using a standardised screening form (see Additional file 2). The investigators will only approach the patient with the agreement of the treating team. As soon as practically possible after this discussion, the study team representative will approach the patient at the bedside to obtain consent. Typically this will be around 48 to 72 hours after the onset of clinical sepsis and the initial collection of blood cultures. For patients with cognitive impairment secondary to their illness (for example, intubation and ventilation, delirium), consent will be obtained from a legally appropriate representative (for example, spouse), where local regulatory requirements allow recruitment of cognitively-impaired persons to clinical trials.

### Study preparation and logistics

The trial coordination centre comprises the project management team (THB, PH and DLP) at the University of Queensland Centre for Clinical Research in Brisbane. Site initiation and training will be conducted by PH, DLP and THB via web conferencing and/or site visits. Study sites will be contacted regularly via teleconference to discuss any issues and ensure consistent study practices across sites.

### Laboratory studies

All blood culture isolates will be made available to the central trial laboratory at the University of Queensland Centre for Clinical Research. Susceptibility testing, including minimum inhibitory concentrations for key antimicrobials, will be repeated in the central laboratory and interpreted according to EUCAST standards. Additional susceptibility testing for agents not tested at the recruiting site laboratories will also be performed. Phenotypic confirmation of ESBL or AmpC production will be performed using combination disc testing [24]. Beta-lactamase genes will be identified and characterized. Isolates will also be screened for carbapenemase genes and other key antimicrobial resistance determinants. Strain typing will be determined using methods such as semi-automated repetitive sequence-based PCR (rep-PCR) (DiversiLab; BioMérieux, Marcy l’Étoile, France) and multi-locus sequence typing (MLST). Isolates will also be available for whole genome sequencing.

### Ethics

The protocol has been given ethical approval by the Royal Brisbane and Women’s Hospital (ref: HREC/12/QRBW/440), the National Healthcare Group (NHG) Domain Specific Review Board (DSRB) in Singapore (NHG DSRB ref: 2013/00453) and the New Zealand Health and Disability Ethics Committee (Ref: 14/NTB/52).

## Discussion

The quality and extent of evidence to help define optimal treatment for many significant bacterial infections is surprisingly limited. This is especially true for the treatment of resistant Gram-negative bacteraemia, despite being a major issue in daily practice for clinicians across many specialties. In addition to laboratory data and expert opinion, current practice is almost entirely based on retrospective observational studies, usually conducted in single centres with relatively small numbers of patients. Such studies, though informative, are always prone to bias, thus weakening the strength of conclusions drawn for these analyses. To date, there have been no RCTs reported specifically comparing different treatment options for ceftriaxone-resistant Enterobacteriaceae.

There is local support for the conduct of this study. This is highlighted by a recent online survey in which 122 infectious diseases physicians in Australia and New Zealand were asked to rank the research studies of greatest immediate clinical relevance - the proposed trial comparing meropenem and piperacillin-tazobactam was the highest ranked RCT in the field of antibiotic resistance in Gram-negative bacilli [25].

Antibiotic resistance is of tremendous public health importance and has been recently described as potentially the greatest current threat to human health [26]. The MERINO trial has potential significance for two reasons. Firstly, it addresses treatment of a common but serious antibiotic resistance issue that is associated with 10 to 20% mortality [5,18]. Secondly, proving that a carbapenem-sparing antibiotic regimen is non-inferior to a carbapenem will help encourage use of alternatives to carbapenems. Carbapenem resistance is the ‘end-game’ with respect to antibiotic resistance as it eliminates one of our most important antibiotic options and leaves few effective alternative treatments. Evidenced-based strategies to support the clinical management of resistant infections that consider both efficacy and the potential for ‘collateral damage’ of treatment options are therefore urgently needed.

## Trial status

A pilot study based on this protocol has commenced recruitment in both Singapore and Australia in February 2014. The remaining study sites are scheduled to begin enrolment later during 2014 once local regulatory requirements have been completed. It is planned for the pilot phase to be extended to a definitive trial in early 2015. It is aimed that recruitment for the definitive study will be completed by late 2016. Data from the pilot phase will be included in the definitive study analysis.

## Additional files

Additional file 1:
**MERINO Trial Clinical Record Form.**
Additional file 2:
**MERINO Trials Initial Screening Form.**

## Abbreviations

APACHE: Acute Physiology and Chronic Health Evaluation
ASID: Australasian Society for Infectious Disease
BLBLI: beta-lactam/beta-lactamase inhibitor
CRF: Clinical record form
CRN: Clinical Research Network
DMSB: Data Monitoring and Safety Board
DSRB: Domain Specific Review Board
ESBL: extended-spectrum beta-lactamase
EUCAST: European Committee for Antimicrobial Susceptibility Testing
QCTBC: Queensland Clinical Trials and Biostatistics Unit
ITT: intention-to-treat
MLST: multi-locus sequence typing
NHG: National Healthcare Group
PCR: polymerase chain reaction
QCTBC: Queensland Clinical Trials and Biostatistics Unit
RCTs: randomised controlled trials
RR: relative risk
