# Supplementary material for: Meropenem versus piperacillin-tazobactam for definitive treatment of bloodstream infections due to ceftriaxone non-susceptible Escherichia coli and Klebsiella spp (the MERINO trial): study protocol for a randomised controlled trial
Source: Trials. 2015 Jan 27;16:24. doi: 10.1186/s13063-014-0541-9 (PMC4311465; doi:10.1186/s13063-014-0541-9)
Supplement: Additional file 1: — MERINO Trial Clinical Record Form. [file 13063_2014_541_MOESM1_ESM.docx]

Researcher’s name:__________________________

Date completed: / /

Patient Label

**Pilot Randomised Controlled Trial of Meropenem versus Piperacillin-Tazobactam for Definitive Treatment of Bloodstream Infections Due to Ceftriaxone Non-Susceptible *Escherichia coli* and *Klebsiella* spp.**

Date of collection of first positive blood culture (blood culture 1) with *E. coli* or *Klebsiella*:

___/___/___ Time_______

**DEMOGRAPHICS:**

Age at time of first positive blood culture: __________ yrs Sex: Male Female

Race: Caucasian Aboriginal/Islander Chinese Indian Malay Other (specify): _________

Type of Residence (home, nursing home etc.):________________________________________________________

**ACQUISITION:**

Date of hospital admission: ____/____/_____

Hospital of admission: NUH TTSH RBWH PAH Wesley BrisbanePrivate SAWMH Wollongong Westmead PeterMac The Alfred MonashHealth Dandenong TheNorthShore Middlemore

**Hospital-acquired**

**Healthcare-associated *** see data dictionary definitions

**Community-associated**

**RISK FACTORS:**

Presence of a medical device in the 7 days prior to the date of collection of the first positive blood culture:

- Vascular catheter (e.g. PICC, Vascath, CVC, port, PIVC), specify type ______________
- Indwelling urinary catheter

,

Therapy within the 30 days prior to the date of collection of the first positive blood culture:

- Chemotherapy
- Oral or intravenous Corticosteroids
- TNF blocker or other biologic (in the last year)*see data dictionary
- Other immunosuppressive therapy, specify________________________________________
- Radiation therapy

Other Risk Factors:

2. Prior surgery within the 14 days prior to the date of collection of the first positive blood culture:

Y / N

Type __________________

Date / /

**PRESUMED SOURCE OF BACTEREMIA:**

- Urinary tract infection
- Intra-abdominal infection Type__________________________________________
- Line related infection
- Surgical site infection
- CNS
- Mastoiditis
- Pneumonia
- Mucositis
- Musculoskeletal
- Skin and Soft tissue Infection including burns Type ________
- Other; please specify: ____________
- Unknown

**ANTIMICROBIALS STARTED 48HRS BEFORE BACTEREMIA and to time of completion of study:**

| **Antimicrobial** | **Dose/route/frequency** | **Single**  **Dose?** | **Date**  **Started** | **Start**  **Time** | **End**  **Date** | **Last Dose**  **Time** |
| --- | --- | --- | --- | --- | --- | --- |
| ***e.g. cefepime*** | ***1gm iv q12h*** | ***No*** | ***17/10/2014*** | ***2000*** | ***27/10/2014=*** | ***0800*** |
|  |  |  |  |  |  |  |
|  |  |  |  |  |  |  |
|  |  |  |  |  |  |  |
|  |  |  |  |  |  |  |
|  |  |  |  |  |  |  |
|  |  |  |  |  |  |  |
|  |  |  |  |  |  |  |
|  |  |  |  |  |  |  |
|  |  |  |  |  |  |  |
|  |  |  |  |  |  |  |
|  |  |  |  |  |  |  |
|  |  |  |  |  |  |  |

*Up to 48 hours before first positive blood culture. Please order data in chronological order if possible.*

# Is the patient in contact isolation at the time of enrolment? Y / N

**DAILY MONITORING:**

*Start on the day of collection of the first positive blood culture (for7 sequential days)*

| Date | ___/___ | ___/___ | ___/___ | ___/___ | ___/___ | ___/___ | ___/___ | ___/___ | ___/___ |
| --- | --- | --- | --- | --- | --- | --- | --- | --- | --- |
| Tmax |  |  |  |  |  |  |  |  |  |
| Tmin |  |  |  |  |  |  |  |  |  |
| Max Heart rate |  |  |  |  |  |  |  |  |  |
| SBP *lowest* |  |  |  |  |  |  |  |  |  |
| Max resp rate |  |  |  |  |  |  |  |  |  |
| Lowest PCO2 if ventilated |  |  |  |  |  |  |  |  |  |
| WCC |  |  |  |  |  |  |  |  |  |
| Neut. |  |  |  |  |  |  |  |  |  |
| Pressor (y/n) |  |  |  |  |  |  |  |  |  |
| Weight |  |  |  |  |  |  |  |  |  |

**ILLNESS SCORE:**

To be performed on patient data collected 48 hours prior to the date of collection of the first positive blood culture.

If the patient is in ICU at this time perform the APACHE 2 score, plus the Charlson Comorbidity Index

If the patient is not in ICU at this time perform the Charlson Comorbidity Index only

If the patient was an outpatient at this time, data collection should be taken from the variables at admission. The Charlson Comorbidity Index should be performed only.

**Charlson Comorbidity Index**

| **Assigned weights** |  |
| --- | --- |
| **1** | Myocardial Infarct or other Coronary Artery Disease  Congestive Heart Failure  Peripheral Vascular Disease  Cerebrovascular Disease  Dementia  Chronic pulmonary disease  Connective Tissue Disease  Ulcer Disease (PUD)  Mild Liver Disease (Child Pugh A)  Diabetes Mellitus |
| **2** | Hemiplegia  Moderate or Severe Chronic Renal Disease (stage 3 onwards i.e. eGFR or CrCL <60mL/min for > 3 months)  Diabetes Mellitus with end organ damage  Any tumor  Leukaemia  Lymphoma |
| **3** | Moderate or severe liver disease (Child Pugh B & C) |
| **6** | Metastatic Solid Tumour  Acquired Immunodeficiency Syndrome |
| **TOTAL SCORE:** | **/37** |

*See appendix for definitions

**
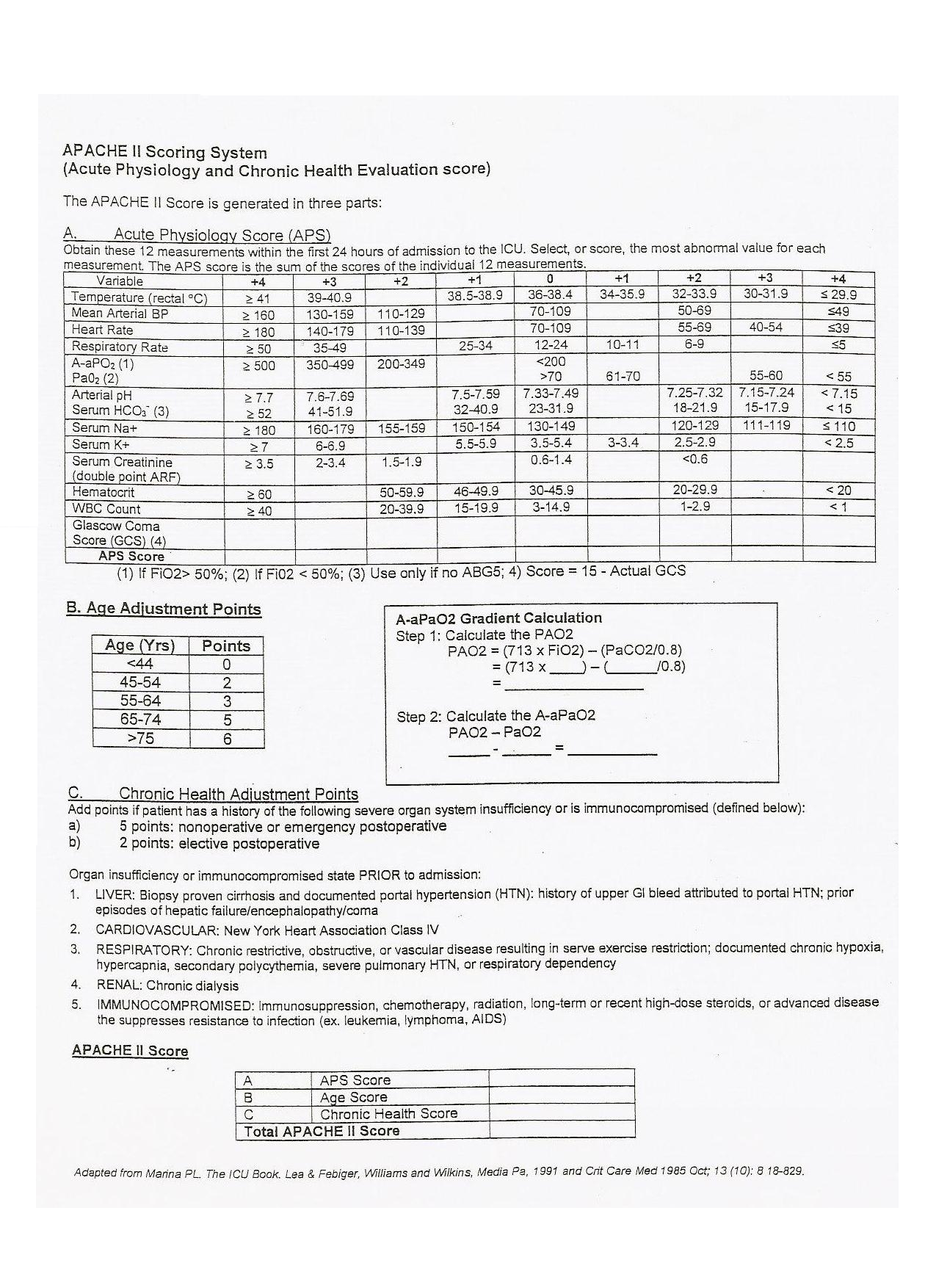
**

**Apache II Score = ____________**

**MICROBIOLOGY:**

Blood Cultures

*Only include one blood culture per day ie if multiple sets taken on a particular day – record 1 set only. If more than 3 days of positive blood culture to be recorded please continue on a new form.*

Blood Culture 1

­­­­­­­­­­­­

Date/time of the culture drawn : ___/___/___, ­­ ___:___

Site of the culture drawn: peripheral other (specify): __________

*O.K. to attach a copy of the susceptibility report instead of filling the table*

| **Antimicrobial** | **S/I/R** | **Antimicrobial** | **S/I/R** |
| --- | --- | --- | --- |
| Amikacin |  | Aztreonam |  |
| Cefepime |  | Ceftazidime |  |
| Ciprofloxacin |  | Colistin |  |
| Gentamicin |  | Doripenem |  |
| Meropenem |  | Piperacillin-Tazobactam |  |
| Ceftriaxone |  | Tobramycin |  |
| Timentin |  |  |  |

Concomitant organisms* on the same blood culture: Y or N

Organism: ________________________

Organism:_________________________

Other sites of growth of GNB within 48 hours before or after the blood culture:

­­­­­ Urine: ________________ CFU/ml

Sputum

Endotracheal aspirate

Bronchoalveolar lavage

Intra-abdominal site, specify: _______________

Central line tip: _________________ CFU

Other: ________________________________________________

Bacteremia Clearance:

*Please list all blood cultures taken culturing E. coli or Klebsiella even if on the same day. Up to 30 days from first positive blood culture; mark + if at least one culture is positive, – if all cultures are negative*­­­­­­­­­­­­-

| Date (MM/DD) | _ /_ /_ | _ /_ /_ | _ /_ /_ | _ /_ /_ | _ /_ /_ | _ /_ / _ | _ /_ /_ | _ /_ / _ |
| --- | --- | --- | --- | --- | --- | --- | --- | --- |
| Time |  |  |  |  |  |  |  |  |
| Blood culture (+ / -) |  |  |  |  |  |  |  |  |
| Pip.-taz. resistant |  |  |  |  |  |  |  |  |
| Mero. resistant |  |  |  |  |  |  |  |  |

Other Positive Blood Cultures:

Y or N

*From 10 days prior to first positive blood culture with E. coli or Klebsiella to 14 days after last positive blood culture*

| DDate Collected | OOrganism1 | Pip.-taz. R  Y/N | Mero. R  Y/N | Organism 2 | Pip.-taz. R  Y/N | Mero. R  Y/N |
| --- | --- | --- | --- | --- | --- | --- |
|  |  |  |  |  |  |  |
|  |  |  |  |  |  |  |
|  |  |  |  |  |  |  |
|  |  |  |  |  |  |  |
|  |  |  |  |  |  |  |
|  |  |  |  |  |  |  |
|  |  |  |  |  |  |  |
|  |  |  |  |  |  |  |
|  |  |  |  |  |  |  |
|  |  |  |  |  |  |  |
|  |  |  |  |  |  |  |
|  |  |  |  |  |  |  |
|  |  |  |  |  |  |  |

1. **Was a meropenem resistant organism isolated from any site at the time of enrolment to 30 days post**

**first blood culture?**

**Yes / No If Yes, specify date___________________ site __________________________**

1. **Was a piperacillin/tazobactam resistant organism isolated from any site at the time of enrolment to**

**30 days post first blood culture?**

**Yes / No If Yes, specify date___________________ site __________________________**

1. **Was Clostridium difficile testing positive from the time of enrolment to 30 days post first positive**

**blood culture?**

**Yes / No If yes, specify date______________________**

**DISCHARGE & IN HOSPITAL MORTALITY:**

# Was a not for resuscitation order placed on this patient during the admission? Y or N

# Date of hospital discharge ___/___/___ or Date of death / /

# Investigator assessment as to whether death was directly due to the *E. coli* or Klebsiella bacteraemia Y or N

# Length of stay post date of first positive blood culture for *E. coli* or Klebsiella _____ days

7 days post first positive blood culture is the patient: alive or dead

30 days post first positive blood culture is the patient: alive or dead
