# Supplementary material for: Meropenem versus piperacillin-tazobactam for definitive treatment of bloodstream infections due to ceftriaxone non-susceptible Escherichia coli and Klebsiella spp (the MERINO trial): study protocol for a randomised controlled trial
Source: Trials. 2015 Jan 27;16:24. doi: 10.1186/s13063-014-0541-9 (PMC4311465; doi:10.1186/s13063-014-0541-9)
Supplement: Additional file 2: — MERINO Trials Initial Screening Form. [file 13063_2014_541_MOESM2_ESM.docx]

Name of screener: __________________________

Current date and time:_________________

Date completed: / /

Patient Label

**MERINO Trial – Initial Screening Tool**

**Please complete on all screened participants so we can**

**determine reasons for exclusion from the study**

1. Must tick yes to all of the following for participant inclusion:

- Blood culture positive for *E. coli* or *Klebsiella*
- Organism is ceftriaxone non-susceptible
- Organism is meropenem susceptible
- Organism is piperacillin/tazobactam susceptible
- Current time is less than 72 hours from the time of the most recent blood culture draw which yielded a positive blood culture **DATE THAT THIS WAS COLLECTED / / Time :**
- Only *E. coli* or *Klebsiella* in blood culture (a single positive blood culture with a skin contaminant is OK – this includes coagulase negative staphylococci, *Bacillus spp, Corynebacterium or Propionibacterium)*
- Patient expected to survive more than 96 hours from now
- Patient not allergic to piperacillin/tazobactam or other penicillins (cephalosporin allergy ok)
- Patient not allergic to meropenem or other carbapenems
- Patient is 18 years or older *(21 years or older for Singapore site only)
- Treatment is with intent to cure this infection
- The patient has not been previously enrolled in this study
- Treating team approval received;
- Consent form signed by participant

Or

- Legal Substitute Decision Maker consent obtained
- **Participant Refused.**

**Reason for Refusal: _______________________________________________________________**

If informed consent gained, proceed to stratification’s (a) and (b).

**(a) Stratification by organism**

Is the organism *Escherichia* *coli*? - Enrol in Stratum E

Is the organism *Klebsiella*? - Enrol in Stratum K

**(b) Stratification by severity of illness**

1. Likely source of infection is the urinary tract: □ YES □ NO

(as indicated by a positive urine culture); if YES BLOCK 1 (no need to calculate Pitt score)

1. Pitt Bacteraemia Score is **4 or less** on the day

first positive blood culture is collected (see scoring below): □ YES □ NO

**The Pitt Bacteraemia Score†**

**Criterion Points**

| **Fever (oral temperature)**  ≤35°C or ≥ 40°C  35.1–36.0°C or 39.0–39.9°C  36.1–38.9°C | 2  1  0 |
| --- | --- |
| **Hypotension**  Acute hypotensive event with drop in systolic blood pressure > 30 mm Hg and diastolic blood pressure > 20 mm Hg  or  Requirement for intravenous vasopressor agents  or  Systolic blood pressure < 90 mm Hg | 2 |
| **Mechanical ventilation** | 2 |
| **Cardiac arrest** | 4 |
| **Mental status**  Alert  Disoriented  Stuporous  Comatose | 0  1  2  4 |
| **Total Score** |  |

* All criteria are graded on the day of first positive blood culture.

If YES answered to (i) or (ii) – Enrol in block 1

If NO answered to both (i) and (ii) – Enrol in block 2

Write study stratification here: Stratum _______ Block _______

Now choose the envelope with that stratum letter and block number, to determine which study drug the patient receives.

Meropenem 🞎 Piperacillin-Tazobactam 🞎
